# Organisational, hygiene- and team-related changes in German general practices during and after the COVID-19 pandemic: a participatory cross-sectional survey among medical assistants (WiSBAH study)

**DOI:** 10.1186/s12889-026-26523-0

**Published:** 2026-02-07

**Authors:** S. Kersten, C. Kersting, A. Mortsiefer, S. Weissbach, A. Calis, N. Berges, E. Hohmann, D. Dehnen, J. Schweizer

**Affiliations:** 1 Institute of General Practice and Primary Care (iamag), Witten/Herdecke University, Witten, Germany; 2 Institute of General Practice and Family Medicine (AM RUB), Medical Faculty, Ruhr University Bochum, Bochum, Germany; 3 Institute of General Practice, Medical Faculty, University of Duisburg-Essen, Essen, Germany; 4 Institute for Digitalization and General Medicine, Medical Faculty, RWTH Aachen University, Aachen, Germany; 5 Institute of General Practice, Medical Faculty, University of Münster, Münster, Germany; 6 North-Rhine Westphalian General Practitioner Research Network NRW-GPRN, Essen, Deutschland

**Keywords:** COVID-19 pandemic, Medical assistants (MAs), General practice, Workload management, Telemedicine, Workplace stress, Hygiene measures, Team interaction, Practice organisation, Mental health

## Abstract

**Background:**

Even before the COVID-19 pandemic, the daily work of medical staff in general practices was characterised by high stress levels; during the pandemic there was a further increase in workload. In response, many practices implemented organisational and hygiene-related measures primarily to maintain patient care and infection control, which also affected working conditions, particularly for medical assistants (MAs). This study aimed to investigate the MAs’ perspective on changes in practice organisation, hygiene, and team interaction that facilitated their work during the COVID-19 pandemic.

**Methods:**

This study was conducted as a cross-sectional study among MAs within the North-Rhine Westphalia General Practitioner Research Network (NRW-GPRN). The questionnaire was developed participatorily in eight focus groups with 50 MAs and refined through cognitive pretesting. Data were collected anonymously via LimeSurvey between August and December 2023. Descriptive analyses were performed in SPSS.

**Results:**

A total of 355 questionnaires were completed by MAs. Almost all (*n* = 343, 96.6%) reported that the GP practice they worked in had introduced telephone consultations at the start of the pandemic; about one third reported implementation of video consultations (*n* = 109). Both measures largely facilitated the work of MAs. A change in internal work processes during the pandemic was reported by 41.1% (*n* = 146) of employees due to changed responsibilities and by 28.5% (*n* = 101) in connection with changed working hours, and changes were partly perceived as a reduction in workload. Additionally, 38.6% reported improved team communication, which further contributed to a reduction in workload. Furthermore, most MAs (60.6%) considered stress reduction measures useful during periods of high workload and named a variety of measures that could help cope with stressful work phases. At the same time, nearly two thirds (63.1%) felt burdened by the increased risk of infection even after the pandemic. Consistent with this, 74.5% (*n* = 264) reported continuing to wear masks when dealing with acutely infected patients.

**Conclusions:**

This study highlights how organisational adjustments, role clarity and team communication were perceived by MAs during and after the COVID-19 pandemic. While the survey does not allow causal inference or assessment of psychological constructs, the findings provide practical insights for improving workload management and supporting MAs in general practice.

**Supplementary Information:**

The online version contains supplementary material available at 10.1186/s12889-026-26523-0.

## Background

Even before the coronavirus disease 2019 (COVID-19) pandemic, the day-to-day work of medical staff in German general practices was characterised by a high workload, frequent interruptions, administrative duties and intensive patient interaction [1, 2]. When COVID-19 emerged, general practices experienced an additional surge in workload. This increase was driven by the high number of acutely ill patients, rapidly changing regulatory requirements and the need to vaccinate large parts of the population within a short time frame [3–6]. Practices also had to adapt organisational structures and hygiene protocols quickly to meet dynamic requirements [6–8].

MAs, who constitute one of the largest non-physician professional groups in ambulatory care in Germany [9], play a central role in ensuring the day-to-day functioning of general practices. They carry out a broad range of clinical, administrative and organisational tasks and act as essential contact points for patients in routine care.

Studies from German primary care have shown that MAs experienced the organisation and documentation of COVID-19 vaccinations, vaccine supply uncertainties and challenging patient interactions as major additional stressors, often associated with overtime, increased emotional strain, a lack of appreciation and feelings of being overwhelmed [10–12].

Even outside pandemic conditions, structural aspects of MAs` work have been shown to be closely linked to job satisfaction and retention. In a large cross-sectional study including 2,371 MAs in Germany, Mergenthal and Güthlin reported comparatively high overall job satisfaction, but low satisfaction with income and perceived appreciation; responsibility and teamwork scored highest [13]. These findings indicate that MAs already worked under demanding conditions before the pandemic and that organisational and structural factors significantly shape their job satisfaction.

Despite their central role, the perspectives of MAs on organisational changes and working conditions remain comparatively understudied, particularly in contrast to the more extensive research on general practitioners or nurses. This gap in evidence limits understanding of how pandemic-related measures affected MAs specifically and where support is needed from their perspective.

At the same time, GP practices in recent years have faced increasing difficulties in recruiting and retaining qualified MAs, with multiple reports highlighting a growing shortage and rising turnover intention among this professional group [14–16]. Understanding how MAs experience their work environment has therefore become increasingly important, as insufficient support may further aggravate existing staffing problems. Particularly during periods of extremely high workload, measures that help reduce strain and strengthen job satisfaction are essential [3, 15–17]. During the pandemic, numerous organisational and hygiene-related measures were introduced to ensure continuity of care. Some of these adaptations have been retained because they proved beneficial in daily practice. Examining how MAs perceived these measures provides valuable insight into which changes may remain useful in routine care.

Existing national and international studies have described substantial adaptations in primary care during the pandemic, including triage systems, telemedicine, infection-specific workflows and modified staffing structures [18–21]. However, most of this research reports on practice management changes or physician perspectives, with far less emphasis on how these measures affected MAs specifically. Beyond pandemic-related work, international research on team-based primary care suggests that expanding the MAs´ role within multidisciplinary teams can increase both workload and job satisfaction. Sheridan et al. found that MAs in U.S. practices transitioning to team-based care reported higher workloads but also greater satisfaction, attributed to stronger relationships with colleagues, more meaningful patient contact and a greater sense of control and efficacy [18]. These findings emphasise that organisational design and team structures can substantially shape MAs´ experiences and perceived working conditions.

However, little is known about how MAs in German GP practices themselves experienced these changes and which aspects they perceived as easing or intensifying their daily workload, and which were maintained beyond the pandemic for this reason. This highlights the need for empirical evidence directly reflecting MAs´ perspectives on organisational, hygiene-related, and team-related measures.

The WiSBAH study was developed participatorily with MAs to address this gap. It focuses on describing organisational, hygiene-related, and team-related measures implemented during and after the pandemic and examines how MAs perceived their impact on daily workload. Rather than applying a theory-driven model in the study design, the study aims to provide empirically grounded, practice-oriented insights into German MAs’ work environments. In this context, “stress” is used pragmatically to refer to work-related strain during high workload periods as described by MAs, and not as a psychological construct measured with standardised scales.

## Methods

This article is structured according to the STROBE statement [19].

### Study aim and objectives

This study was conducted as a cross-sectional online survey. It aimed to describe organisational, hygiene-related, and team-related measures implemented in German general practices during and after the COVID-19 pandemic and to assess how these changes were perceived by MAs with regard to their daily workload. The study was designed to be descriptive and exploratory; no causal inference or measurement of psychological constructs was intended.

### Participatory questionnaire development

An interprofessional working group of the NRW-GPRN organised a kick-off event called “MFA-Forum” in March 2023, which was designed exclusively for non-physician personnel working in German general practices. In total, 50 persons participated. During the two-hour online event, conducted via Zoom *(*Zoom Video Communications, Inc., 2023, Zoom version 5.13.10), these 50 participants took part in eight focus groups. The discussions followed a semi-structured guide including questions on changes in acute patient care, organisational adjustments, perceived helpful or unhelpful measures, and potential contributors to job satisfaction.

Based on the answers collected during these discussions, the interprofessional working group identified the most relevant topics for the questionnaire. As no validated instruments were available that adequately assessed the topics identified, a customised, German-language online questionnaire was designed on the basis of Porst’s question wording rules [20]. The final questionnaire was then transferred into LimeSurvey, enabling online distribution via a web-based link.

The final questionnaire addressed three main topics and comprised 24 core items (Additional File 1):

- Practice organisation (14 items).
- Hygiene and distancing measures (4 items).
- Team interaction (6 items).

Different response formats were used depending on the content of the item. For most attitudinal statements, we applied a 4-point Likert scale where indicated [1] ‘strongly disagree,’ [2] ‘mostly disagree,’ [3] ‘mostly agree,’ and [4] ‘strongly agree,’ with an additional option ‘don’t know.’ For organisational measures (e.g. changes in responsibilities or working hours), participants were first asked whether a measure had been introduced in their practice (yes/no/don’t know) and, if applicable, to rate its impact on daily practice on a 5-point scale ranging from “rather more difficult” to “made things much easier”. Dichotomous items (e.g. whether telephone or video consultations were offered) used yes/no/don’t know response options.

In addition, the questionnaire asked for demographic data and information on the participants’ professional qualifications. Free-text fields enabled respondents to detail specific changes and provide individual feedback. For the open-ended item on stress-management measures, participants could mention up to four measures. This item was included because MAs had emphasised in the focus groups that coping with high workload phases was a relevant issue in daily practice. It therefore captured perceived usefulness and practical suggestions rather than implementation or effects of specific interventions. These free-text responses were inductively coded into six categories (relaxation techniques, team-building activities, working-time and break regulations, internal communication strategies, recognition and appreciation, financial benefits) and then summarized descriptively. The survey questionnaire is available as an additional file [Additional file 1].

In order to identify comprehension difficulties, the questionnaire was piloted with five MAs using the Think Aloud method [21]. Feedback on clarity, structure, and visual presentation was incorporated, and revisions were made iteratively until no further adaptations were required.

### Setting and recruitment

Data collection finally took place from August 30, 2023, to December 31, 2023.

Recruitment used multiple dissemination channels that were open in parallel throughout the entire survey period. Each channel received its own LimeSurvey link, to facilitate distribution. The survey link was distributed via a training course for MAs, mailing lists from research networks and universities, social media channels of the North Rhine Association of Statutory Health Insurance Physicians (KVNO), and the Association of Medical Assistants (VMF e.V.). Details on the number of respondents included in the analysis and their self-reported recruitment pathways are reported in the Results section.

### Data management

All questionnaire data were stored within the LimeSurvey system hosted at the University of Duisburg-Essen. After survey closure, data were exported and transferred to IBM SPSS Statistics for Windows, Version 29.0 (IBM Corp.)

To ensure alignment with the study aim, we included only respondents who self-identified as MAs and reported currently working in a GP practice. Questionnaires from MAs working in specialist practices and from non-MAs were therefore excluded from analysis. Incomplete questionnaires were excluded only if one or more of the central survey items (organisational, hygiene-related or team-interaction items) were missing. Items on demographics and professional background were optional and could therefore contain single missing values. These missing values were not systematic and did not affect the analysis of the primary survey items.

Missing data occurred in the optional demographic variables (e.g. age, years in job, employment status, practice location). Because inclusion required complete responses to all core survey items, these demographic missing values did not affect the analysis of primary outcomes. Responses differ across items due to conditional display logic and item-specific non-response; all descriptive analyses were based on valid cases per item.

### Data analysis

For continuous variables, means and standard deviations (SD) were calculated; for categorical variables, frequencies (n) and percentages were reported. All analyses were descriptive, in line with the study’s exploratory aim. Subgroup analyses (e.g., age, practice location, duration of employment) were examined but not conducted due to insufficient statistical power and the absence of meaningful differences. No inferential statistics or multivariable models were performed.

### Ethics and consent

All participants were informed about the purpose of the study, voluntary participation, and the anonymity of their responses. Informed consent to participate was given by opening the questionnaire. The Ethics Committee of the Medical Faculty of the University of Duisburg-Essen (23–11354-BO; 23.08.2023) approved the study.

## Results

### Participant characteristics

Across all channels, 639 individuals accessed the survey. Of these, 355 respondents met the eligibility criteria and provided complete data for inclusion in the final analysis. Participants were asked how they became aware of the survey. Among the 355 respondents included in the analysis, more than half learned about the study through the Association of Medical Assistants (VMF e.V.) (*n* = 193; 54.4%). Internal dissemination via HAFO.NRW and universities accounted for 18.0% (*n* = 64), followed by channels of the North Rhine Association of Statutory Health Insurance Physicians (KVNO) (*n* = 51; 14.4%) and other external dissemination pathways such as professional mailing lists or training-related distribution (*n* = 47; 13.2%).

The mean age of the participants was 42 years, with a range from 19 to 71 years (24 missing values). On average, the participants had been working for 19 years. Table 1 summarises all socio-demographic characteristics; responses vary across items due to optional demographic questions.

**Table 1:** Socio-demographic characteristics of study participants (*n* = 355)

Characteristics (*n*= responses in total)	*n* (%)
Age (*n* = 331)	19 to 71 years, mean 42 years
Sex (*n* = 355)	
Male	2 (0.6)
Female	353 (99.4)
Years in job (*n* = 332)	
Average	19 years, mean 19.0, median 19.1, SD ± 10.5 years
Less than 10 years	69 (20.8)
10–29 years	199 (59.9)
More than 30 years	64 (19.3)
Employment status (*n* = 353)	
Full-time (> = 37.5 h/week)	189 (53.5)
Part-time (< 37.5 h/week)	164 (46.5)
Practice location (*n* = 352)	
Urban (more than 20,000 inhabitants)	174 (50.6)
Rural (fewer than 20,000 inhabitants)	178 (49.4)
Level of qualification (*n* = 354)	
Medical assistant (MA)	328 (92.7)
MA additional qualification VERAH/NaePa (*n* = 328)	105 (32.0)
Nurse	16 (4.5)
Practice is part of a research network (*n* = 352)	62 (17.6)

### Changes in GP practice organisation

Of the 355 participants, the vast majority (*n* = 343; 96.6%) implemented telephone consultations. Among those 88.6% (304 of 343) perceived telephone consultations as easing their daily work. Approximately one third (*n* = 109; 29.9%) introduced video consultations. Among those practices reporting introduction of video consultations, 106 respondents provided a valid rating; of these, 63.2% (67 of 106) perceived video consultations as easing their daily work.

Changing responsibilities were reported by 41.1% (*n* = 146) of respondents. Of those, 45.2% (66 of 146) indicated that these changes eased daily practice. Examples included the introduction or modification of tasks related to a fixed division of labor, such as telephone or e-mail services. Almost one third (*n* = 101, 28.5%) reported changes in working hours. Specific changes, detailed in a free-text field, included shift work, adjustments in working hours, and changes in break times. Of the 101 respondents, 24.8% (*n* = 25) reported longer working hours/overtime, 7.9% (*n* = 8) shift work, 10.9% (*n* = 11) special consultation hours and changes in break management, and 5.9% (*n* = 6) changes in team organisation and coordination. Nearly three quarters of the participants reported (*n* = 255; 71.8%) that their GP practice established new infectious disease consultation hours. About two-third of MAs (151 of 255; 59.2%) described this change in practice organisation as easing daily practice, while 21.7% (*n* = 77) reported an increased workload.

### Hygiene and distancing measures

**Fig. 1:**
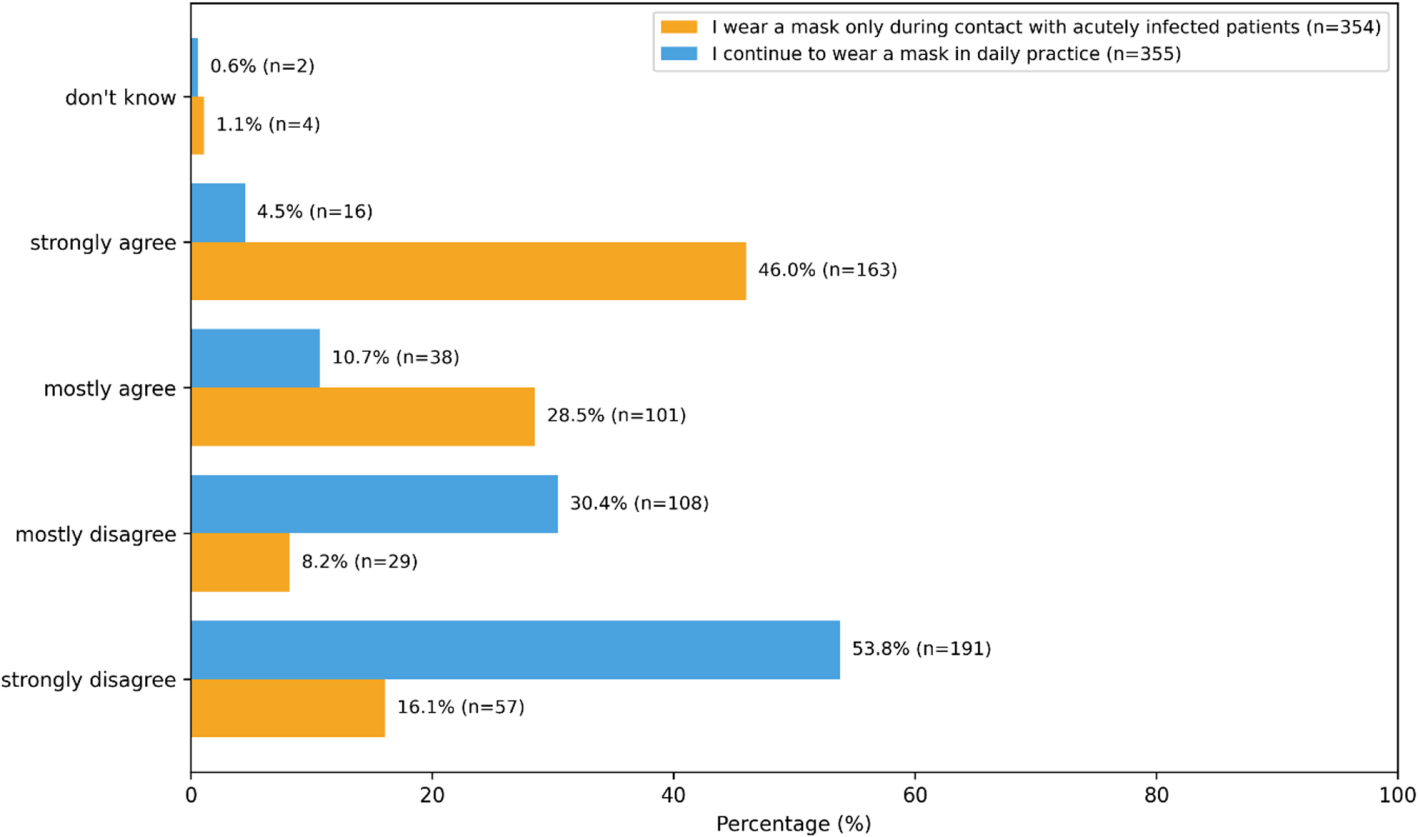
Mask use and distancing measures reported by MAs in GP practices

Regarding infection risk, 63.1% (*n* = 224) of MAs agreed or strongly agreed that they still felt additionally burdened by the risk of infection in daily practice after the end of the pandemic (Fig. 1). 15.2% reported that they generally continue to wear a mask in daily work, whereas 84.2% disagreed with this statement. In contrast, almost three quarters indicated that they only wear a mask when in contact with acutely infected patients. Mask use was assessed through three separate Likert-scale items. Figure 1 shows the full distribution of Likert responses for each mask-related item. A total of 259 participants (73.0%) agreed (fully agreed *n* = 208,58.6%, mostly agree *n* = 51, 14.4%), practices should maintain distancing measures such as a plexiglass screen.

### Team interaction, recognition by manager, stress management measures

According to 38.6% (*n* = 137) of MAs, the pandemic led to improved team communication. Among respondents with valid ratings on this item 80% (*n* = 107) perceived improved communication as easing the workload (‘mostly agree to ‘strongly agree’). At the same time, 50.2% (“strongly” or “mostly disagree”) did not perceive an improvement in team communication and 11.3% responded “don’t know”.

For respondents who did not report improved communication, it is not possible to judge whether team communication was already good before the pandemic or whether no positive change occurred.

**Fig. 2:**
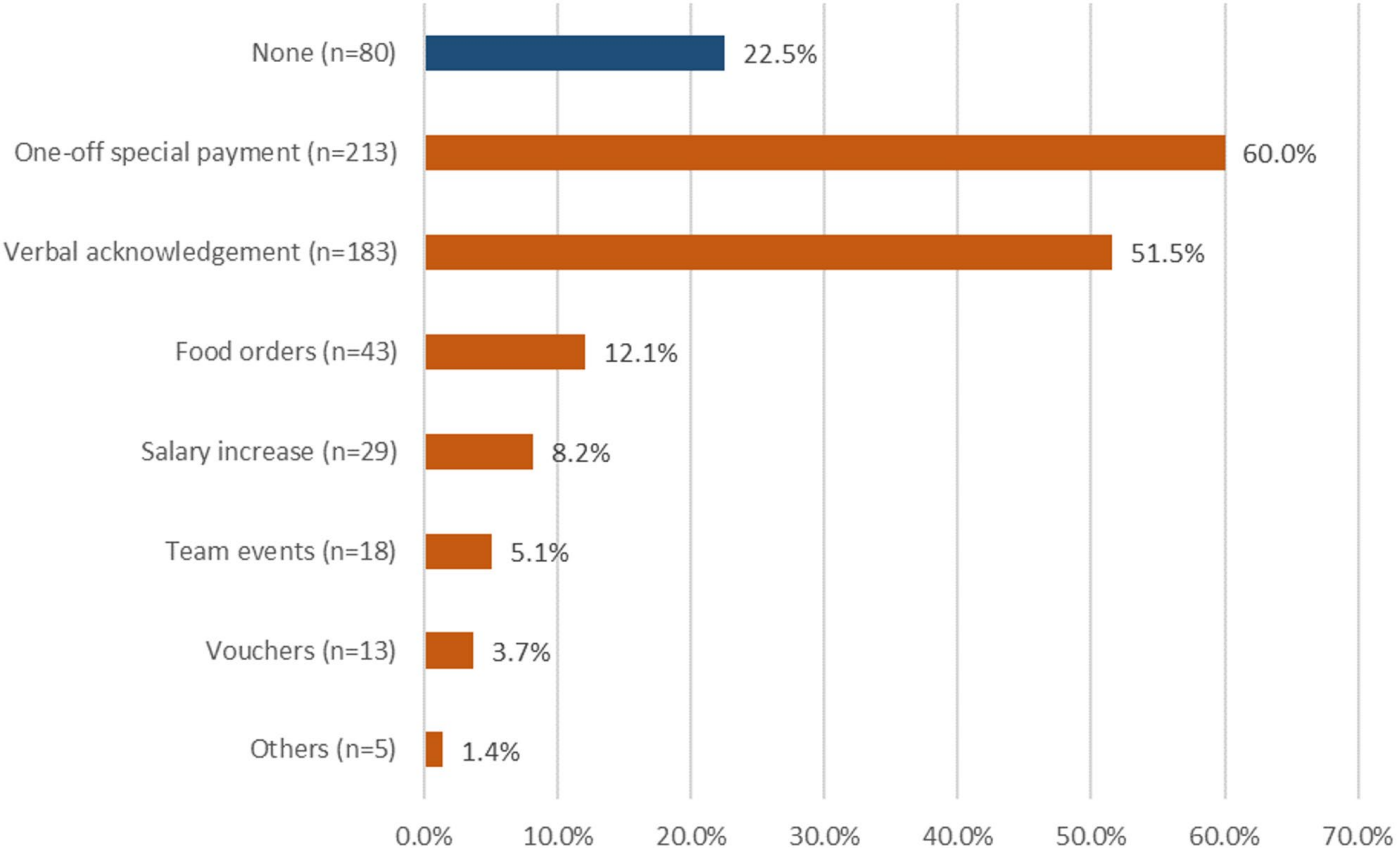
Types of recognition from medical practice management during the COVID-19 pandemic (multiple answers possible; *n *= 355)

Figure 2 displays the forms of recognition received from medical management. The majority received a tax-free one-off special payment (‘corona bonus’), while more than a fifth received no recognition within the practice. Slightly more than half reported verbal appreciation.

**Fig. 3:**
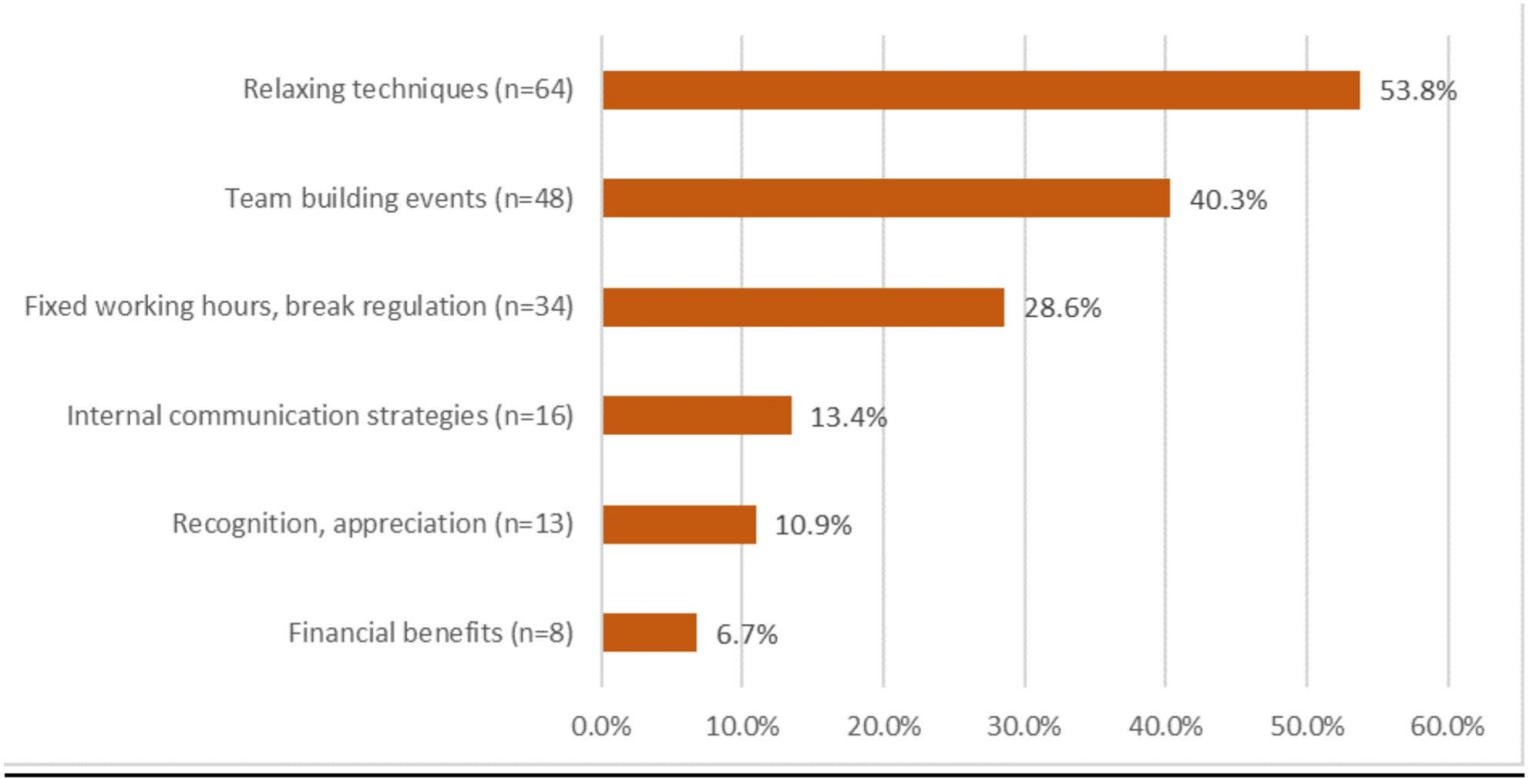
Categories of measures suggested by MAs to cope with stressful work phases (multiple answers possible; *n* = 119)

According to most MAs, measures for stress management are a useful concept in times of high workload (*n* = 215, 60.6%), while 106 MAs (29.9%) disagree and 34 MAs (9.6%) are unsure. The item captured perceived usefulness rather than the implementation of concrete stress-management activities. MAs reported a bundle of measures that could help to cope with stressful work periods (Fig. 3).

Table 2 summarises the above-mentioned changes in the workplace and evaluates these measures from the perspective of the MAs.

**Table 2:** Organisational, hygiene-related and team-related measures implemented during the COVID-19 pandemic and their perceived impact on daily workload (percentages refer to valid responses per item)

Measure	Implemented *n* (%)	Perceived reduction in daily workload *n* of those implemented (%)
Introduction of Telephone consultations	343 (96.6)	304 (88.6)
Introduction of Video consultations	109 (29.9)	67 (63.2)
Changes in responsibilities	146 (41.1)	66 (45.2)
Changes in working hours	101 (28.5)	27 (26.7)
Establishing infectious disease consultation hours	255 (71.8)	151 (59.2)
Distancing measures (e.g., plexiglass)	259 (73.0)	-
Improved team communication	137 (38.6)	107 (80.0)
Stress management measures perceived as helpful	215 (60.6)	-

## Discussion

This descriptive, exploratory study among 355 MAs with an average working experience of 19 years revealed substantial organisational, hygiene-related, and team-related adjustments in German general practices during and after the COVID-19 pandemic. Overall, the findings illustrate how practices adapted their workflows quickly and pragmatically to maintain patient care under crisis conditions. Because the study was designed as a participatory, descriptive survey, these results provide insight into how MAs perceived specific measures rather than allowing conclusions regarding psychological constructs such as resilience, burden or long-term coping capacity.

Despite their indispensable role in GP practices, there is currently little literature on the views and experiences of MAs in German GP practices. In view of an increasing shortage of MAs and an increasing trend among MAs from practices to jobs away from patients [22, 23], more consideration should be given to the needs of MAs.

### Adaptability of general practices

A number of studies have revealed challenges faced by practices due to the COVID-19 pandemic: poor communication with the authorities, changing regulations, bureaucratic hurdles, lack of recognition, financial losses, fear of infection, lack of protective equipment, and pressure on patient care. Without clear guidelines, practices often relied on self-developed measures to manage these shortcomings [24].

The WiSBAH study shows that practices adapted well to pandemic changes by adjusting working hours, reorganizing practice workflows, and increasing telemedicine use to safeguard patient and staff safety. This is in line with other studies from Switzerland, the Netherlands and Australia, where organisational measures and flexible working models were also implemented swiftly and successfully [25–27]. However, an important distinction must be emphasised: many of these measures were introduced primarily to maintain patient care under pandemic conditions, not to actively reduce the workload of MAs. Their workload-reducing effects were therefore secondary and unintended. This aligns with qualitative findings from Germany [10], which showed that MAs perceived vaccination-related organisational measures as necessary but burdensome, and with international evidence showing that rapid organisational adaptation in primary care often shifted additional coordination tasks to support staff [7, 28]. Rawaf et al. reported that in many countries, practice teams had to reorganise workflows overnight, extend remote care pathways and develop improvised patient-flow systems with minimal external guidance, also often resulting in substantial additional organisational burden for non-physician staff [7].

This underscores that the organisational burden carried by MAs in German GP practices is not an isolated phenomenon, but part of a broader international pattern in which primary care teams absorbed system-level gaps during the COVID-19 pandemic.

### Telemedicine

The introduction of telemedicine, including telephone and video consultations, was a significant measure. Nearly all practices (96.6%) used telephone consultations on the basis of a regulation that allows doctors to issue sick notes by phone [29], with the aim of reducing workload and protecting staff [24, 30, 31]. This regulation extends in Germany beyond the pandemic since December 2023 [32].

In contrast, fewer practices (29.9%) offered video consultations, likely due to administrative efforts, inadequate reimbursement, and unclear usage guidelines [33]. Concerns about internet connectivity and low acceptance among older patients may also have contributed to this [33, 34]. However, most MAs found both telephone (88.6%) and video consultations (63.2%) to be workload-reducing.

While these findings indicate perceived benefits, telephone and video consultations should not be interpreted primarily as workload-reduction strategies but rather as pandemic-driven service adaptations, which may have had secondary positive effects on workload.

Compared with international data, adoption patterns were similar: studies from the USA and the Netherlands reported rapid telehealth expansion, with support staff taking on substantial new coordination tasks.

Despite concerns, practices need to adapt to video consultations due to the push for healthcare digitalisation. Benefits of telemedicine, such as direct patient interaction and time savings, should be emphasised. It has the potential to save costs and resources, reduce workload, and increase job satisfaction within the practice team, especially among MAs. However, training for the practice team is necessary to address uncertainties and concerns.

### Changes in responsibilities and work organisation

Many practices reorganised their workflows internally, introducing fixed responsibilities for tasks like phone and email services, changing working hours with extended shifts, shift work, and new break regulations. New measures also included infection consultation hours and special workdays like “vaccination Saturdays.”

Over 40% of the surveyed MAs felt that fixed responsibilities reduced their workload. Fixed responsibilities likely reduce the number of competing tasks and interruptions, which is particularly relevant for MAs who coordinate a large share of patient communication and practice organisation.

Changes in responsibilities can also be interpreted within the Job Demands-Resources model (JD-R) [35] which distinguishes between work-related demands and resources and their relevance for perceived workload and well-being. From this perspective, changes in task allocation may represent a combination of demand reduction and resource enhancement. Clear task allocation may reduce cognitive and organisational demands by limiting interruptions, ambiguity and role conflicts, while simultaneously increasing employees’ sense of structure and control. In this study, nearly half of the respondents experiencing changes in responsibilities perceived these as workload-reducing, underlining the practical relevance of this mechanism. Establishing transparent and stable task allocations can therefore represent a low-threshold organisational strategy for alleviating high workloads in general practice teams.

A quarter (28.5%) favored changes in working hours, likely due to the impact on their personal lives.

Infection consultation hours were viewed as “(hardly) made things (quite/much) easier” by slightly more than half (59.2%) of respondents, while 40% found them “rather more difficult”, possibly due to increased patient discussions, shortened breaks or overtime, as mentioned in the questionnaire.

Effective organisational restructuring in GP practices requires well-planned measures that seamlessly integrate into the daily routines of GP practice staff, thereby reducing workload. Adjusting tasks, responsibilities, and implementing flexible working hours can significantly alleviate perceived strain experienced by MAs.

### Cultural change in handling infections

One important finding, for which there is little evidence to date, is the persistent perceived burden of infection risk among MAs. More than half continued to feel burdened by the risk of infection even after the pandemic, and a large proportion said they continued to wear masks when treating infectious patients.

From a JD-R perspective, persistent concerns about infection risk represent job demands that are largely non-modifiable in primary care settings. Such enduring demands cannot be eliminated, but require compensation through adequate organisational and social resources. The continued use of protective measures, despite the end of formal pandemic regulations, suggests a sustained orientation towards safety that can be understood not as resistance to change, but as an adaptive response to ongoing job demands. Practical implications include the continued prioritisation of clear hygiene protocols, regular training and transparent risk communication, rather than an uncritical return to pre-pandemic routines.

The continued use of masks and distancing measures such as plexiglass screens reported by MAs is significant and points to a long-term cultural shift in infection management in primary care. This topic is one that merits specific investigation, particularly with regard to perceived safety and risk communication in practices.

Because in addition to the high workload, the fear of infection also increases stress for the MAs [3, 11]. Our findings are consistent with other studies showing that health concerns, ongoing stress, and uncertainty about patient care are risk factors for burnout and stress-related health problems such as depression and anxiety [36].

### Team interaction, recognition and stress management

Few studies have focused on team interaction from the perspective of MAs in general practices. In the joint development of our study, MAs emphasized the importance of team spirit and communication. This includes interactions among MAs and between MAs and GPs.

Contrary to our expectation of improved communication in times of crisis, only a little more than a third of respondents reported improved communication (38.6%) within the team during the pandemic, while about half did not perceive an improvement and a further 11.3% were unsure. It remains unclear whether some practices may have already had efficient communication structures in place before the pandemic and therefore no further improvement was recorded. Those who experienced improved communication also perceived this as easing their workload (80%). Within the JD-R model, improved team communication and perceived recognition can be understood as key social and organisational job resources. Although only a minority of MAs reported improved communication during the pandemic, those who did overwhelmingly perceived this as workload-reducing. This finding suggests that even limited improvements in communication and recognition may have a substantial buffering effect on high job demands. From a practical perspective, this highlights leadership behaviours, regular feedback and structured communication routines as feasible levers for supporting MAs in everyday practice. Other studies also cited poor communication, low social interaction, and a lack of appreciation as psychological stress factors, and emphasised the importance of social support and participation [6, 36–38]. These contribute to coping with high workloads, maintaining job satisfaction, and having a protective mental effect. In addition to improved communication structures, recognition comes in various forms. The WiSBAH study showed that, in Germany, MAs often received tax-free bonuses (“corona bonuses”), praise, and words of appreciation from practice owners. Sustainable salary increases, meal orders, or team events were less common. Around a quarter (22.5%) of respondents stated that they did not receive any additional recognition. Whether this reflects an already appreciative work environment or a lack of acknowledgement cannot be derived from these data.

In line with other studies, the data suggest that stress management, a balanced workload, and good social interaction are essential for a healthy working environment, mental well-being, and job satisfaction [36, 39, 40]. It is important to emphasise that the items on stress-management measures in our study capture hypothetical usefulness rather than actual implementation. In our sample, around 60% of MAs considered such measures useful in periods of high workload and mentioned strategies such as relaxation techniques, Yoga, Pilates, joint breaks, team events and timely overtime compensation as potentially helpful. Thus, in keeping with other studies, we can conclude that stress management, a balanced workload, and good social interaction seem to be essential for a healthy working environment, mental well-being, and job satisfaction. In line with the JD-R model, these organisational and social resources (e.g. clear task allocation, supportive team communication, recognition and stress-management offers) may help buffer high job demands [35]. The JD-R model was originally developed as a generic framework to explain occupational strain, motivation and well-being across professions by distinguishing between job demands and job resources. According to the model, high job demands increase the risk of strain and burnout, whereas job resources have motivational potential and can buffer the negative effects of high demands on employee well-being. While our study does not measure outcomes such as burnout or turnover intention, the pattern of our findings suggests that strengthening such resources could be a relevant target for practice development. Accordingly, complementary measures such as active listening and recognizing the needs of employees should also become part of the work culture. These recommendations do not refer to specific techniques measured in this study, but represent practical examples of how the empirically identified resources of supportive team communication and perceived recognition could be strengthened in everyday practice. This interpretation is based on the finding that improved team communication and perceived appreciation were strongly associated with reduced perceived workload among MAs. Particularly in view of the increasing shortage of staff, these measures could help to retain staff in the practices and reduce their migration to other areas.

MAs occupy a unique position in German general practices because they combine clinical activities with extensive organisational and coordinative responsibilities. They manage a substantial proportion of patient communication, appointment triage and workflow organisation, functioning as a key interface between physicians, patients and the administrative structures of the practice [2]. Consequently, organisational measures such as clear task allocation or improved team communication may be particularly impactful for MAs working in General Practices. Some findings may be transferable to other outpatient care professions involved in workflow coordination, but the organisational burden carried by MAs suggests that these patterns are especially salient in this group [23].

### Strengths and limitations

The WiSBAH study has several strengths. A key strength is its participatory approach, which ensured that the survey addressed topics relevant for MAs. The focus on the COVID-19 pandemic provided practice-based insights into a period of exceptionally high workload and the study examined multiple dimensions of working conditions, including organisational changes, hygiene measures and team interaction. This allows for a broad descriptive overview of how MAs perceived practice adaptations. The use of an online questionnaire enabled efficient data collection across Germany and facilitated standardized analysis.

However, the study has notable weaknesses. Voluntary participation could lead to self-selection bias, limiting generalisability. Because recruitment relied heavily on professional networks and the VMF e.V., a considerable proportion of participants may have been particularly engaged or professionally active MAs, which may affect response patterns. The VMF e.V. is the national professional association for MAs, providing continuing education and representing professional interests. Furthermore, the proportion of participants with advanced qualifications such as VERAH/NaePa was comparatively high, which may additionally limit representativeness because these MAs tend to be more experienced and professionally engaged than the broader MAs´ workforce. Only fully completed questionnaires were analysed, which ensured completeness of core survey data but may have excluded partially completed questionnaires that could have provided additional nuance. The sole use of descriptive statistics limits the analytical depth, as no subgroup analyses or causal relationships could be explored. The exclusive reliance on an online survey may have underrepresented groups with limited digital access or lower digital literacy. Despite a broad sample, it cannot be assumed to be representative of all MAs in Germany.

Furthermore, the JD-R model was applied retrospectively as an interpretative framework and was not incorporated in the development of the questionnaire or data collection. This limits the possibility of systematically assessing theoretical constructs. However, the model was used cautiously to contextualise selected findings and to derive practice-oriented considerations, without implying causal relationships.

## Conclusions

The WiSBAH study describes which organisational, hygiene-related and team-related measures were implemented in German general practices during and after the COVID-19 pandemic and how MAs perceived their impact on daily work. Various measures were implemented in GP practices to respond to increased workloads and infection-related strain. According to participating MAs, particularly helpful measures included telephone and video consultations, reducing physical patient flow and infection risks, as well as clear task responsibilities and infection-specific consultation hours, which helped structure workflows and patient management.

Viewed through the JD-R model, these findings can be interpreted as indicating that, while some pandemic-related job demands persist, such as ongoing concerns about infection risk and increased organisational complexity, organisational and social resources such as clear task allocation, supportive communication and recognition may help buffer these demands. In this sense, the model provides a useful interpretative framework for understanding why certain measures were perceived as particularly helpful by MAs, without implying causal relationships.

To increase job satisfaction, MAs suggested flexible work models, visible appreciation and recognition, improved team communication, stress management support, and adequate personal protective equipment with regular training. These suggestions align with international research and underscore the importance of organisational and social resources for maintaining well-being and retention among practice staff.

Overall, the findings highlight the importance of sustaining supportive organisational measures to improve working conditions and job satisfaction among MAs. Given the increasing shortage of qualified MAs in Germany, such measures may contribute to stabilising the workforce and reducing turnover in general practice.

## Supplementary Information

Supplementary Material 1.

## Data Availability

The datasets used and analysed during the current study are available from the corresponding author upon reasonable request.

## Abbreviations

COVID-19: Coronavirus disease 2019
DEGAM: German Society of General Practice and Family Medicine (Deutsche Gesellschaft für Allgemeinmedizin und Familienmedizin)
GP practices: General practitioner practices
KVNO: North Rhine Association of Statutory Health Insurance Physicians
MAs: Medical assistants, nurses working as MA
NaePa: Non-medical practice assistant (Nicht-ärztliche/r Praxisassistent/in)
NRW-GPRN: North Rhine-Westphalian GP Research Practices Network
VERAH: Health Care Assistant (Versorgungsassistent/in in der Hausarztpraxis)
VMF e.V.: Association of Medical Professionals
JD-R model: Job Demands-Resources model
