# Supplementary material for: Organisational, hygiene- and team-related changes in German general practices during and after the COVID-19 pandemic: a participatory cross-sectional survey among medical assistants (WiSBAH study)
Source: BMC Public Health. 2026 Feb 7;26:716. doi: 10.1186/s12889-026-26523-0 (PMC12931044; doi:10.1186/s12889-026-26523-0)
Supplement: Supplementary file 1 — Supplementary Material 1. [file 12889_2026_26523_MOESM1_ESM.pdf]

Strategies for Managing High Workload Among Medical Assistants in German General Practices During and After the COVID-19 Pandemic: Findings from the WiSBAH Study

Dear medical assistants,

The COVID-19 pandemic has led to a significant increase in workload in GP practices. In this challenging situation, the team at the HAFO.NRW GP Research Practice Network is interested in your experiences:

We would like to understand the strategies you have employed to better cope with this workload and what measures have worked well in terms of practice organisation.

Your participation in this anonymous survey is very important, as it will actively contribute to to optimizing practice organization and easing future workloads in daily practice.

Please note that your participation is voluntary. You are free to end the survey at any time without this having any consequences. The survey is completely anonymous, and no personal identifiers will be linked to you or anyone else. No registration is required to participate in the study.

Confidentiality and data protection:

The data retention period is 10 years after analysis is completed. All data will be deleted after this period. All persons involved in the project are subject to professional confidentiality and are obliged to maintain data protection.

If you have any questions about the use and processing of your data, please contact
Susanne Kersten
Email: Susanne.Kersten@uni-wh.de

Witten/Herdecke University, Chair of General Practice II and Patient-Centredness in Primary Care, iamag, Institute of General Practice and Primary Care (iamag), Phone: 02302-926 7194.

We sincerely appreciate your participation. You can start the survey here:

Organisational measures that have led to changes in daily practice routines during the COVID-19 pandemic:

Insured persons who were unable to work due to a mild respiratory illness were able to take sick note for up to 7 days until 31 March 2023 after taking a medical case history during the telephone consultation by the doctor.

In the following you will see some statements about your medical practice and your work, which you should rate with one cross for each question.

1. **the possibility of issuing an sick note by telephone was offered by our practice.**

❒ Yes ❒ No ❒ Don´t know

2. **issuing a sick note by telephone has made everyday practice life easier.**

❒	❒	❒	❒	❒
Strongly disagree	Somewhat disagree	Somewhat agree	Strongly agree	Don´t know

3. **it should also be possible in future to issue a sick note by telephone for trivial illnesses (minor flu-like infections, gastrointestinal infections, etc.).**

❒	❒	❒	❒	❒
Strongly disagree	Somewhat disagree	Somewhat agree	Strongly agree	Don´t know

More video consultations were offered during the COVID-19 pandemic.

1. **<u>video consultations were offered in our practice</u>.**

❒ Yes ❒ No ❒ Don´t know

2. **the video consultation has made daily practice routine easier.**

❒	❒	❒	❒	❒	❒
Strongly disagree	Somewhat disagree	Somewhat agree	Strongly agree	Don´t know	No video consultations offered

Organising the daily practice routine:

The following organizational measures were introduced or changed in our practice during the COVID-19 pandemic (please tick the applicable statement):

1. **change of tasks/responsibilities (e.g. fixed work responsibilities, telephone/email service)**

   ❒ No, not introduced

   ❒ Don´t know

   ❒ Yes, has been introduced:

The change in tasks/responsibilities has made daily practice routine …

❒ Rather more difficult	❒ Not changed	❒ barely easier	❒ Quite a bit easier	❒ Made things much easier

2. **change in waiting areas (e.g. separate areas for patients with appointments and patients with infections/acute illnesses)**

   ❒ No, not introduced

   ❒ Don´t know

   ❒ Yes, has been introduced:

The change to the waiting areas has made daily practice...

❒ Rather more difficult	❒ Not changed	❒ barely easier	❒ Quite a bit easier	❒ Made things much easier

3. **change in working hours (break times, shift work, opening hours over lunchtime, etc.)**

   ❒ No, not introduced

   ❒ Don´t know

   ❒ Yes, has been introduced:

The change in working hours has made daily practice …

❒ Rather more difficult	❒ Not changed	❒ barely easier	❒ Quite a bit easier	❒ Made things much easier

❒ if „yes, has been introduced“ was entered - free text for the question: which specific change to working hours was implemented? ___________________

4. **Newly introduced/extended infection/acute consultation hour**

❐ No, not introduced

❐ Don´t know

❐ Yes, has been introduced:

The newly introduced/expanded infectious/acute consultation has made the daily practice routine...

❐	❐	❐	❐	❐
Rather more difficult	Not changed	barely easier	Quite a bit easier	Made things much easier

5. **Appointment-only consultation hours (for all patients, including infectious/acute patients)**

❐ No, not introduced

❐ Don´t know

❐ Yes, has been introduced:

Appointment-only consultation hours for all patients have made everyday practice …

❐	❐	❐	❐	❐
Rather more difficult	Not changed	barely easier	Quite a bit easier	Made things much easier

Hygiene and distancing measures

During the COVID-19 pandemic, the legislature ordered additional hygiene measures for medical practices.

1. **Even after the end of the COVID-19 pandemic, I feel additionally burdened by the risk of infection in daily practice life.**

❐	❐	❐	❐	❐
Strongly disagree	Somewhat disagree	Somewhat agree	Strongly agree	Don´t know

2. **I continue to wear a mask (face mask/FFP2) in daily practice:**

❐	❐	❐	❐	❐
Strongly disagree	Somewhat disagree	Somewhat agree	Strongly agree	Don´t know

3. **I only wear a mask (face mask/FFP2) when in contact with acutely infected patients.**

❐	❐	❐	❐	❐
Strongly disagree	Somewhat disagree	Somewhat agree	Strongly agree	Don´t know

4. **The measures for distancing from patients at the reception of the practice, e.g. in the form of a plexiglass screen, should be maintained for my sense of safety.**

❒	❒	❒	❒	❒
Strongly disagree	Somewhat disagree	Somewhat agree	Strongly agree	Don´t know

Team interaction

The COVID-19 pandemic often led to changes in team interaction.

1. **the pandemic has led to improved team communication.**

❒	❒	❒	❒	❒
Strongly disagree	Somewhat disagree	Somewhat agree	Strongly agree	Don´t know

2. **the improvement in team communication has made work easier.**

❒	❒	❒	❒	❒
Strongly disagree	Somewhat disagree	Somewhat agree	Strongly agree	Don´t know

3. **during the pandemic, I have received more recognition from the medical practice management.**

❒	❒	❒	❒	❒
Strongly disagree	Somewhat disagree	Somewhat agree	Strongly agree	Don´t know

4. **During the pandemic, I received the following recognition for the additional workload from the practice management (multiple crosses are allowed here!):**

❒ Verbal acknowledgement
❒ Food orders for the team
❒ Team events
❒ Vouchers e.g. for massage, restaurant, etcne z.B. für Massage, Restaurant, etc.
❒ One-off special payment (so-called “Corona bonus”)
❒ Salary increase
❒ Others:__________________________
❒ None
❒ Do not know

5. **In times of high workload, measures/offers for stress management are useful (e.g. relaxation training, joint breaks, coaching).**

❒ Strongly disagree	❒ Somewhat disagree	❒ Somewhat agree	❒ Strongly agree	❒ Don´t know

❒ **Which measures do you consider to be included:** _______(free text)

We would now like to ask you to provide some additional information about yourself:

Year of birth: ____(four-digit number possible)

Geschlecht: ❒ female ❒ male ❒ diverse

I have the following professional qualification (multiple crosses are allowed here!):

- ❒ Medical assistant in apprenticeship
- ❒ Medical assistant
- ❒ Medical-technical assistant (MTA, also MTLA, MTRA etc.)
- ❒ Nurse or nursing specialist
- ❒ Physician Assistant
- ❒ Other, namely the following: _____(free text)
- ❒ Without professional qualification
- ❒ Don't know

My additional professional qualifications (multiple crosses are allowed here!):

- ❒ None
- ❒ Don't know
- ❒ VERAH (Health Care Assistant)
- ❒ NäPa/EVA (non-medical practice assistant)
- ❒ Certified specialist for outpatient medical care
- ❒ Practice management
- ❒ Other: ______ (free text)

I am currently working in a GP practice

- ❒ Yes
- ❒ No (please state your specialist title) ________ (free text)

I am working full-time

- ❒ Yes
- ❒ No, please state weekly working hours ________ (free text)

My professional experience in a GP/specialist practice (in years, excluding apprenticeships):

_____________years

❒ Don´t know

The practice where I work is located in a:

- ❒ Rural community
- ❒ Small town (≤20.000 inhabitants)
- ❒ City (≤ 100.000 inhabitants)
- ❒ Large city (> 100.000 inhabitants)
- ❒ Dont´t know

Please enter the first three digits of the zip code of your practice location: _________ (free text)

The practice where I work is part of a research practice network

- ❒ HAFO.NRW
- ❒ SaxoForN
- ❒ RAPHAEL
- ❒ FoPraNet
- ❒ BayFoNet
- ❒ RESPoNsE
- ❒ Other research practice network:s _____(free text)
- ❒ No
- ❒ Don´t know

Wie haben Sie von der Umfrage erfahren?

❒ MFA-Forum

❒ HAFO.NRW

❒ An Institute for general practice

❒ Other: _____(free text)

Is there anything else you would like to tell us that we have not yet asked?

____________ (free text)

Together we can gain valuable insights that can help to improve working conditions in general practices and address the challenges more effectively.

You can find out about the current status of the study at the website HAFO.NRW

Thank you in advance for your participation in this important study!
